# A Review of the Types of Training Aids Used for Canine Detection Training

**DOI:** 10.3389/fvets.2020.00313

**Published:** 2020-06-05

**Authors:** Alison Simon, Lucia Lazarowski, Melissa Singletary, Jason Barrow, Kelly Van Arsdale, Thomas Angle, Paul Waggoner, Kathleen Giles

**Affiliations:** ^1^AGS Forensics, LLC, Washington, DC, United States; ^2^Canine Performance Sciences, College of Veterinary Medicine, Auburn University, Auburn, AL, United States; ^3^Federal Bureau of Investigation Laboratory, Quantico, VA, United States; ^4^Giles Consulting, LLC, Huntington Beach, CA, United States

**Keywords:** training aids, canine detection, pseudos, terminology, non-pseudo alternatives

## Abstract

The canine detection community is a diverse one, ranging from scientific fields such as behavior, genetics, veterinary medicine, chemistry, and biology to applications in law enforcement, military, medicine, and agricultural/environmental detection. This diversity has allowed for a flourishing and innovative community, yet it has also led to little acceptance and agreement on terminology. This is especially true when discussing the variety of training aids used in olfactory-based exercises. In general, authentic materials and pseudo-scents are the most commonly discussed, with the former accepted widely for training and certification, and the latter more often disregarded. However, as advances are made in the creation of training materials, alternative training aids are being introduced that do not fit into either of these categories. The misconceptions surrounding how these alternative training aids are manufactured has led to confusion on their classification, and therefore their reliance as an effective tool. This manuscript will review the existing language surrounding canine training aids, address relevant research revealing effectiveness, and clarify the different types based on their manufacture, chemical nature, and fundamental function.

## Introduction

Target substances in canine detection are exceedingly varied, ranging from traditional materials such as narcotics, explosives, human scent, and human remains, to less common or emerging targets such as diseases, pests, and wildlife. This diversity of targets is mirrored by the professional community. Opinions, research, and experience from canine handlers and trainers, behavioral sciences, genetics, veterinary medicine, and analytical sciences, as well as various organizations and government agencies, influence the training methods and protocols of canine teams. This wealth of information has made the canine community inventive and successful. Yet, there is a considerable lack of agreement across the community regarding a standard terminology. Such disparity complicates effective transfer of knowledge across the canine industry, impeding advancements in technology and methodologies.

The variety of jargon specific to the canine community is especially apparent when referring to types of training aids. Training aids can be created by the onsite trainer, by an assisting specialist (such as a bomb technician), or in a laboratory. Such aids differ based on their manufacture, chemical nature, and fundamental function, yet this specific information is rarely discussed, is often proprietary, and has limited third-party evaluation or support. Thus, there is often confusion regarding what to call certain categories of training aids. The purpose of this article is to discuss current existing jargon, and to define them based on their function and chemical nature.

There are two assumptions made in this article regarding terms and definitions. First, the terminology of *odor* and *scent* used herein are derived from the Organization for Scientific Area Committees (OSAC) Dogs and Sensors subcommittee (1). OSAC is an organization administered by the US National Institute of Science and Technology (NIST), which replaced the Scientific Working Group for Dogs and Orthogonal detector Guidelines (2), and makes standards and guidelines for the canine detection community. *Odor* refers to the “volatile chemicals emitted from a substance that are able to be perceived by olfaction,” while *scent* refers specifically to the “volatile chemicals emitted from a live human” (1).

Second, the article will focus specifically on sources of odor and scent rather than odor delivery systems or transport containers. The odor/scent source is the training aid itself, or the object providing the target odor/scent. Odor delivery systems are devices that contain the training aid, such as “scent” boxes, the Mixed Odor Delivery Device (MODD) (3), the Training Aid Material Delivery Device (TAMDD) (4), or the Training Aid Delivery Device (5), for example. Transport containers are used to “move training aids in compliance with storage and handling guidelines of the Federal, state, and/or local agencies' policy” (1).

**Figure 1:**
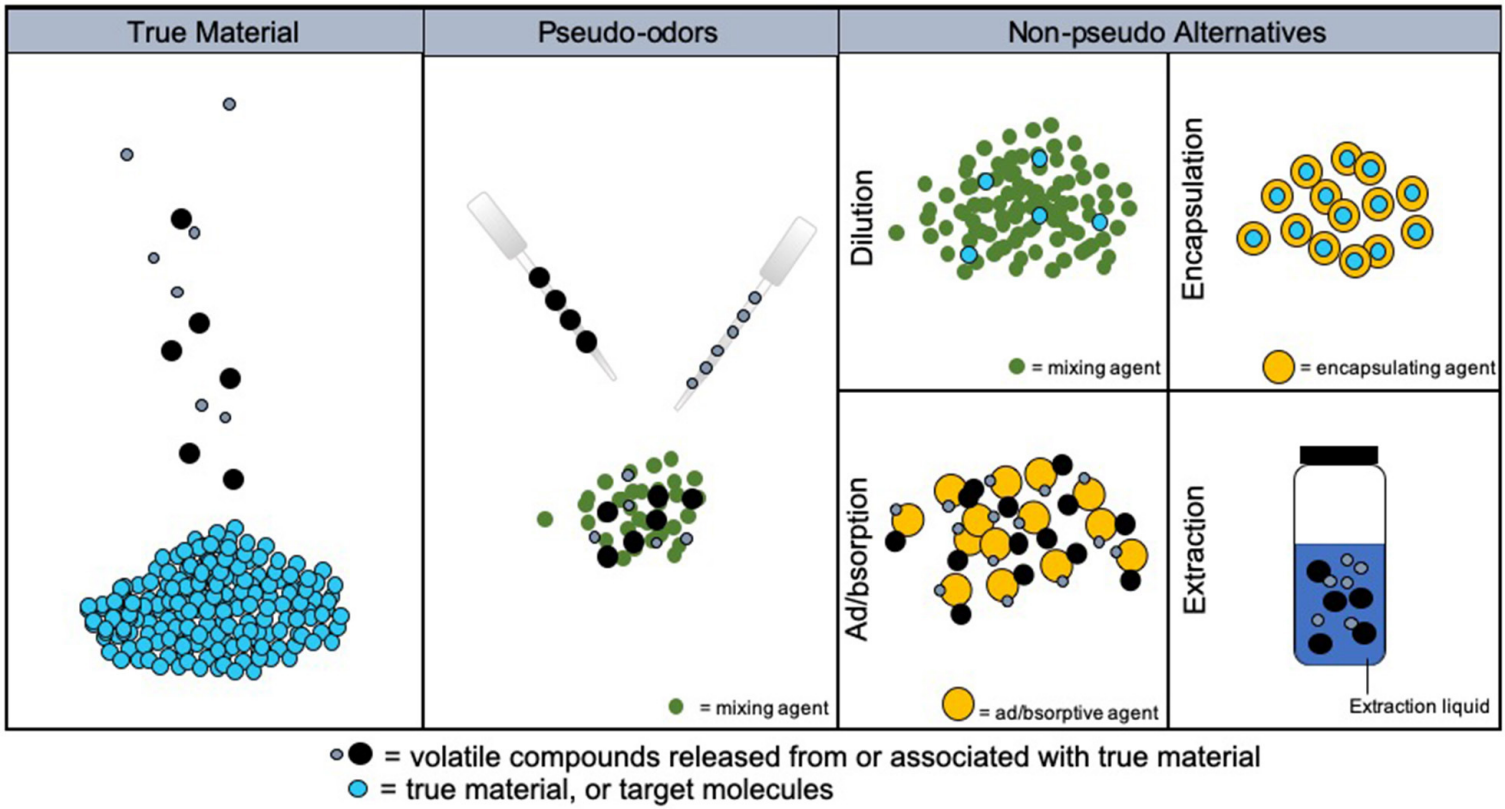
Visual representation showing the chemical and manufacture differences between true material, pseudo-odors, and non-pseudo alternatives, given the same true material.

Considering these assumptions, the following discusses three categories of training aids as determined by different methods of manufacture: true material, pseudo-odors, and non-pseudo alternatives (see Figure 1). True material is the actual target substance. Pseudo-odors are created in a way so that the true material has no direct part in their manufacture. Non-pseudo alternatives are made through utilization of the true material in their manufacture. Each of these sources of odor/scent are examined in detail below, along with discussions of their function. We will also examine any existing research which sheds light on their efficacy and accuracy as training aids.

## True Material

True material, also referred to as bulk material, actual material, genuine material, or parent material, refers to the target substance itself, whether it be an explosive, a narcotic, human remains, or any other target. True materials may be in a solid [e.g., composition-4 (C4) or cocaine], liquid [e.g., nitromethane or ethylene glycol dinitrate (EGDN)], or gaseous (e.g., human scent or certain chemical warfare agents) phase. For solid and liquid true materials, canines generally locate the source of the odor, whereas for gaseous true materials, they may simply be identifying the presence or absence of the odor/scent. These substances are currently what is recommended best practice for use in training and certification (2), though, as will be discussed, other types of training aids may serve as suitable training materials. However, it is unlikely that true material will be replaced in certifications, since those records are necessary for most law enforcement and military organizations where canine searches may result in probable cause for entry or evidence collection.

While the use of true materials may seem straightforward, there are actually many considerations to be made in the selection of these materials. It is generally accepted that training dogs on the purest form of a substance is the best method for ensuring reliable detection. However, training on a single pure odor has been shown to produce a strong response and subsequent detection to that specific odor while narrowing the tendency to respond to variations of it (6). This degree of specificity presents a challenge due to the high variability in targets that a dog may encounter, such as homemade explosives or improvised explosive devices that often consist of mixtures of various compounds. Recent publications suggest that in addition to the pure odor, the inclusion of additional mixtures in training could improve a canine's ability to generalize in other contexts of background or conflicting odors. For example, studies by Lazarowski et al. (7) and Hall and Wynne (8) each found that canines trained to detect ammonium nitrate (AN) utilizing only a pure source did not proficiently [to the Military Working Dog criterion of 95% success (9)] locate mixtures containing AN. However, when mixtures were included in training scenarios, the canines' proficiency in detecting other AN-based mixtures increased (8). Even when considering AN apart from mixtures there is some debate as to whether industrial grade or laboratory grade should be considered purer. DeGreeff et al. (10) analyzed the volatiles from seven different sources of AN, and found different quantities of ammonia and contaminates were present, depending on the manufacture method and form of the AN (i.e., prill or ground). These studies speak to the difficulty of selecting a pure training material that will lead to the greatest success of the canine team. While it is folly to assume that these same patterns will be observed in all true target materials, there is existing literature that identifies differences in either canine behavior or the chemical odor or scent profiles for various pure materials: human remains (11–13), human scent (14–16), blood (17, 18), potassium chlorate mixtures (19–21), trinitrotoluene (TNT) (22, 23), hexamethylene triperoxide diamine (HMTD) (24, 25), single- and double-based smokeless powders (26, 27), marijuana (28, 29), synthetic cathinones (30), and methamphetamine (31), among others. However, there is also literature implying that for certain highly volatile compounds, canines readily perceive variants of a true material as the same, for example nitromethane (32), triacetone triperoxide (TATP) (33) and accelerants (34).

While the selection of a true material may seem complex based on this information, it is often directed by departmental, state, or federal regulations, or based on operational needs. Depending on the legality of obtaining, maintaining, and transporting a substance, certain forms of that substance may be regulated to a point that prohibit a team from training on certain forms. This issue is often encountered, for example, by human remains detection teams. It may not be possible to obtain visually identifiable remains in some states within the United States, and it is not possible to obtain any human remains for training purposes in many countries. Therefore, many handlers turn to human teeth or pig remains as surrogate training materials (35, 36). While human teeth may be a portion of human remains, there is ample chemical evidence that these odors differ from other types of human remains (11, 36).

As another example of limited choice for true materials, safety of an explosive, toxic, or infectious material can limit access. Most explosives teams within the United States train on peroxides provided by a single federal entity. The peroxide HMTD has been shown to have extremely variable odor profiles depending on synthesis and age (24, 37). Yet, for safety purposes, a single, high purity synthesis route is followed for the creation of HMTD canine training aids. This means that despite drastic differences in the spectrum of HMTD odor profiles, there is actually only a single option for a true HMTD training aid. Toxic hazards are also tightly regulated, often limiting certain narcotics training aids, such as fentanyl or synthetic cathinones, to seized material. Such materials can have high purity, depending on the seizure, but it does limit greatly the choice of synthesis route and manufacturing method that may be desired for certain operational requirements. For example, in one study of synthetic cathinones, canines trained to detect seized samples were successful in locating another bath salt [seized α-pyrrolidinopentophenone (α-PVP) and ethylone were each used to imprint a group of canines, (38)]. However, bath salt formulations vary greatly by region, so this may not hold true in all jurisdictions.

Viruses, bacteria, diseases, pests, agriculture, and wildlife/conservation, which are growing applications for canine detection, have other complications to consider. These target materials are exceedingly varied based on the age of the disease, infection, or agricultural product, leading to wide variability in the range of odor profiles that the dog needs to be able to detect. Some diseases have periods of time when a person, animal, or plant is infected with a virus or bacteria, but not yet ill or displaying symptoms. Plus, training aids are often presented in the context of extremely high background, for example in fecal samples, or background that could be very interesting to a dog's natural abilities and desires, such as urine. Such background odors or scents could confuse the actual target, and complicate generalization to other sources of the target odor. Each of these considerations are key when selecting which true material will be used for imprinting the odor and for training in operational scenarios, when even more extraneous background odors/scents may be encountered, such as an outdoor environment at a port or a grove, or even detection in a live species. There are a handful of manuscripts describing choices and chemical analysis of true material for such detection purposes: for cancers (39, 40) and other medical alerts (41), agriculture (42), and wildlife or pests (43, 44). Of course, safety and regulation can also limit these choices. Infectious materials can be tightly regulated to help stint the spread of diseases, limiting training with the true material to a specific geographic area. As another example, bed bug detection has become a common application of canines, yet the age of the bugs can influence true material choices. Further, keeping the bugs or insects from spreading is imperative when training, even though no specific regulations exist, which may influence the choice of age. One recent study evaluating training aids for the detection of an insect pest in Australia found that canines trained using dead insects successfully found the live counterpart on the majority of the test trials (45). However, the sample size was small with a limited number of observations and the authors noted that such generalization may not occur with other, non-specialized and less experienced canines.

In addition to selecting a true material with which to imprint, train, and certify, there are considerations to take into account in the maintenance of the material, which can alter its odor/scent profile and affect the way a canine perceives it. While the age of a substance is of particular importance for such living true materials, it is also key for other materials. For example, chemical analysis of HMTD over time has shown how unstable the compound is. The odor profile changes drastically due to natural decomposition of the molecule (24, 25, 37). This is also the case for other compounds, such as TNT, AN, or cocaine (10, 46, 47).

A related consideration is how often target materials should be varied in training. For example, human scent is unique to each individual (48). Yet, if a canine team does not alter their true material (in this case a human decoy or target), then the canine may learn to identify only that person. It has been shown that canines associate positively with familiar human scents, even when the familiar scent is not their handler (49). The tendency to associate a scent with an individual person creates a particular challenge in the training of disease detection. For example, dogs trained to detect prostate cancer apparently memorized the set of training samples rather than the common cancer odor profile, as their detection dropped when tested with samples from new individuals (50). This memorization of people as targets is a learned behavior, and an issue that can be remedied by varying the target material so that memorization becomes too difficult and learning the common odor class is a more efficient strategy for the canine. A similar issue may arise outside the subdiscipline of human scent and arises from handling. If only one person handles the material for all training sessions for a substance, the canine may associate that person's scent with the true material. While this is also a learned behavior, it relates more to a lack of experimental controls than to the target material itself. However, if the target is not varied, then that single person's scent will remain on the target and will continue to be associated with the target's odor profile. It is also worth noting that these inadvertent clues caused by familiar human scent are separate from other handler influences that may evolve in the course of canine training, such as handler stress or handler beliefs (51, 52).

As another example of varying the true material, contamination and cross-contamination can occur if materials are stored too closely together. In a study from 1997, Hallowell et al. (53), found that canines trained to detect nine separate explosives using true material could only identify the most volatile explosive species. The true materials were all stored in the same explosives bunker, and cross-contamination confused the odor profiles. These examples all help to demonstrate that even when using true materials, there are many considerations that must be made to ensure that the true material is actually representative of the desired target substance, and is therefore an effective training material.

## Pseudo-odors

True material, while generally considered best practice, is not always available due to cost, handling, storage, transportation, safety, and security challenges (54). Therefore, several types of alternative training aids have been developed, one of which is referred to as pseudo-odors, “pseudos,” odor mimics, or simulants. “Pseudo” is a term that is often used in the canine community, yet is infrequently applied correctly or consistently. In general, a pseudo training aid is one in which the true material had no part in its manufacture. The most common method for making a pseudo training aid is to identify the major chemical components in the headspace of the true material, and use pure (or neat) compounds to create a physical mixture of those components intended to simulate the odor profile. One technical magazine review separated these training aids into four types based on which chemical components were included in the training aid: active odorant, byproduct or impurity, filler or additive, and a non-related volatile that attempts to mimic the perceived smell of the target (55). As will be discussed, the pseudos composed of active odorants tend to be more supported in the published literature; however, there is a lack of published information regarding many proprietary pseudos, and these remain unverified by an independent evaluator.

There are pros and cons to the pseudo approach in training. A clear advantage to using pseudos is they can be used as a training material when access to the true material is regulated, whether for safety or legal reasons. They can therefore provide access to substances to a greater number of canine teams. For example, a study that used sarcosine as a target molecule for canines trained to detect prostate cancer observed sensitivity and specificity to sarcosine that was comparable to the detection of prostate cancer in urine (56). The use of such a pseudo-odor could improve canine detection training for medical purposes by easing access to training materials and increasing their uniformity. However, there are many problems associated with pseudos as well. The most prominent disadvantage is associated with complex target materials, such as human remains and HMTD. As discussed above, such true materials change odor profiles dramatically with time, environment, and storage conditions. This makes creating accurate pseudo training aids very complicated. From the most basic perspective, the presence of varied odor profiles corresponding to the target means that one combination of neat chemicals does not truly represent the various odor profiles of the true material. Additionally, pseudos often produce much more odor than the original material, which could provide a chemical hazard to the canine. The amount of odor could also affect the canines' perception of that trained odor, influencing threshold. For example, dogs trained to detect a certain quantity of an explosive do not necessarily respond to larger or smaller quantities of the same material. Further, the material used to contain the chemicals, generally cellulose, diatomaceous earth, low-density polyethylene (LDPE) bags, or similar materials, also produce odors that could change perception of the odor, especially if blanks or negative controls are not provided. Blanks and negative controls in this instance refer to the use of packaging materials within a training session to help proof (or proof off) of extraneous materials. Many of these challenges are also applicable to non-pseudo alternative training aids, as will be discussed.

The unsuitability of multiple pseudo training aids has been shown both chemically and in canine behavioral studies. Studies of several narcotic and explosive pseudo training aids, plus human decomposition pseudos have demonstrated that these substances are not generally successful in canine trials. For example, one study examining the effectiveness of pseudo training aids for single-based smokeless powder, TNT, and C4 showed that the pseudos tested were poor simulations of the true material, despite containing previously-identified explosive-related odors in their headspace. Canines were trained on either the pseudo or the real explosive and then tested on the counterpart. Alert rates to the test odor were well below proficiency, ranging from 0 to 25%, indicating that the dogs did not perceive the real material and the pseudos interchangeably (57). Further evaluations of pseudos for pentaerythritol tetranitrate (PETN), hexogen (RDX), TNT, chlorate, and nitrate had similar results: none of the pseudos were effective in training dogs to detect the true material (26, 27). Heroin and marijuana pseudos have also shown ineffective. Only one out of 12 trained detection canines alerted to an acetic acid-based heroin pseudo, and no canines detected a marijuana pseudo, suggesting that the dogs perceived the pseudo as different than the true material they had been trained with (27). Rice and Koziel (58) supported these findings when they used odor activity values to examine one commercial brand's pseudo materials for heroin, marijuana, and cocaine, and determined that they do not adequately represent the odor impact of the true material. Human decomposition pseudos have been analyzed for chemical and behavioral response similarity to true material, and none of the existing pseudos (at the time of the research) were efficient odor mimics (59, 60). Such publications make the defense of pseudos in court difficult, which could invalidate an otherwise legal search or seizure.

There are several possibilities for why pseudos fail to perform in the same way as true material. Pseudos make significant changes to the original odor profile through the dilution, absence, or addition of chemical substances. These inconsistencies mean that commercial aids may not provide the same volatiles as the true material, or they may alter volatile ratios (27). Further, because pseudos are created using pure chemical compounds, these compounds must be held in place with some mixing agent, such as cellulose or diatomaceous earth, which could change the transportation properties associated with how the molecules enter the atmosphere. Without considering these physical properties, any complete comparison of a pseudo material to the material it is attempting to imitate is not possible. Finally, the pure chemicals are usually also combined in a way that provides a much larger quantity of odor than what would be encountered with the true material, which could affect canine perception of the material and could also be hazardous.

While the efficacy of pseudos has been largely disputed by scientific analysis, there are some cases where a pseudo is useful and scientifically shown to be accurate, both by chemical analysis and canine behavior. The best example of this is for cocaine. Cocaine, depending on the synthesis and manufacturer, can produce a variety of chemicals in the odor profile, such as methyl benzoate, benzoic acid, methyl cinnamate, anhydroecgonine methyl ester, trans-cinnamic acid, and ecgonine methyl ester. However, canine analyses from two independent research groups have shown that canines identify methyl benzoate as the active odorant of cocaine. In other words, canines alert to the presence of methyl benzoate, a decomposition product of cocaine, rather than the cocaine molecule itself (47, 61, 62). Further, canine alerts to cocaine have been upheld as proficient for probable cause in the Florida State Supreme Court, even given the evidence surrounding methyl benzoate as an active odorant suitable for training. In the case of *Florida v. Jardines*, the defense argued that because canines detect methyl benzoate, and methyl benzoate is also a product of snapdragon flowers and perfumes, that canines are not specific enough for an alert to serve as probable cause. Subsequent research and the final court decision ruled in favor of the canine alert (63, 64). While methyl benzoate is the most established pseudo in the detection community, there are others with published evidence of support. For example, piperanol as a pseudo for 3,4-methylenedioxymethamphetamine (MDMA) elicited a canine alert rate of 60% (65). There is equal support for the use of 2,4-dinitrotoluene (2,4-DNT) (50% alert rate) and 2-ethyl-1-hexanol (66.7% alert rate) for TNT and C4, respectively (46). While these are not operationally proficient success rates, they do show that more investigation is warranted.

## Non-pseudo Alternatives

To address the disadvantages of pseudos, while still providing safe access to training aids, many types of non-pseudo alternative training aids have been manufactured. They vary based on the type of target as well as the chemical nature and fundamental function of the aid. Generally speaking, a non-pseudo alternative is a training aid in which the true material had a part in its manufacture, but is not present in bulk. In other words, the true material is utilized to render a safe target through various methods. These training aids are manufactured through four main methods: (1) dilution of the true material by simple mixing, (2) encapsulation of the true material inside another substance, (3) ad/bsorption of the odor of the true material, and (4) extraction of the odor from the true material. Dilution and encapsulation methods still contain trace or small amounts of the true material, while ad/bsorption and extraction of the odor do not.

### Dilution

Dilution refers to taking small, or trace amounts of a target material, and mixing it with larger amounts of an inert solid or dissolving it in an inert liquid. This makes the true material safe by lowering the amount of material present, and separating the molecules from each other to remove shock sensitivity, for example. Dilution has been successful anecdotally, but no available reports have evaluated this method scientifically for accuracy or safety. For example, some HMTD training aids available on the market mix HMTD precursors with diatomaceous earth to create a final trace mixture that is non-detonable (66, 67). However, it has been shown that the synthesis method for HMTD has tremendous effect on the resulting odor profile. Without evaluation of the diatomaceous earth method to show any differences or similarities in odor profile to other methods, this method of synthesis may not be comparable. Other dilution matrices may include cellulose, glass beads, or any myriad of other materials.

The most common liquid dilution is water. This method is not commercially available, and is often done in-house. This means it is diverse, and its reproducibility has not been evaluated for each target material. The water-dilution method has been used for many years to help lower absolute threshold for hard-surface tracking of humans. A person's sweaty shirt or hat will be soaked in an un-specified amount of water. The water-scent mixture can then be continuously diluted and sprayed as a trail for the canine to follow. This method has had much anecdotal success; however, it does require a person to spray the trail, so there is generally a secondary, stronger scent trail to follow. The other most common liquid-dilution method is much more recent, and was developed by the Royal Canadian Mounted Police (RCMP) for fentanyl (68). A small amount of fentanyl is dissolved in water, dropped onto a cloth or cotton pad, and allowed to evaporate off. The canine is then trained on whatever fentanyl remains on the cloth. Again, while this method has anecdotal support, the danger exists that it has not been evaluated to determine how much fentanyl remains on the cloth for detection, which means it has not been examined for toxicity.

While the dilution method is common for human scent and many explosives and narcotics, matrix effects are not often considered. Generally, when a training aid manufacturer or canine trainer finds one dilution method that is successful, they tend to continuously use that matrix for all target materials. However, each target substance will have their own unique chemical properties that influence its interactions with that matrix. Such chemical properties as vapor pressure, diffusion rates with the material, polarity, molecular weight, and rates of evaporation or sublimation will affect how quickly a substance diffuses from the matrix. Further, these properties will determine how accurately the odor profile of the dilution training aid simulates that of the bulk material. Continuing with the HMTD example above, HMTD dissociates at room temperature. The diatomaceous earth-based training aids changed the way that the HMTD entered the atmosphere and resulted in poor representation of the bulk material (69). This same consideration must be given to liquid dilutions. For example, fentanyl is only moderately soluble in water. It is therefore plausible that very little material actually enters the liquid mixture, leaving an unknown amount in solution. Dilution training aids can provide easy-to-make non-detonable and non-toxic training aids, yet there is much research that needs to be done to prove their validity.

### Encapsulation

Encapsulation of the target material is similar to dilution in that it places a trace amount of material within a matrix. The main difference is simply the mechanism (i.e., dilution or encapsulation) through which this is achieved. Existing encapsulation devices are mainly for explosives and narcotics, such as HMTD. One method for HMTD and TATP is to encapsulate the peroxide explosive inside microspheres. Heating the microspheres during canine training then releases the odor (70). This method does render the explosive safe; however, it lacks the same matrix consideration as the dilution method. Interactions with the matrix may change the odor profile so that it does not accurately reflect bulk material (69).

### Ad/bsorption

Ad/bsorption of the odor of a target material onto a secondary material is another method of rendering safe a hazardous substance. Simply put, a secondary material, such as steel, cotton, or a polymer, is exposed to the headspace of a true material for a period of time to ad/bsorb the odor profile. The odor is subsequently released over time. This method has been used for many years informally to help lower absolute threshold or to make a hazardous substance easier to transport. For example, Dutch canine trainers have used steel tubes to collect human scent for scent-identification line-ups. This method does allow for easy cleaning of the collection matrix, but it may also provide uneven collection of the target material, depending on how well the steel adsorbs certain chemicals associated with human scent. In some way, this is the same method used in article search training, when a person leaves scent on an object by briefly touching the article.

Cotton and similar natural fibers are probably the most commonly used matrices to absorb odor. It is used in human scent and decomposition odor collection, in both static and dynamic ways. Static collection occurs when the cotton is left near the true material to collect the odor. Dynamic collection occurs when human scent or decomposition odor is pulled through the cotton, using a STU-100 or a similar device (71, 72). While cotton is the most common and reproducible fiber used to collect and release human scent, many other fibers such as cotton-blend, rayon, and wool are effective. Polyester has proven ineffective for the purpose (73, 74). While no canine trials were performed, investigations have been made into the static collection of target volatiles for C4 and single-based smokeless powder onto cotton gauze, with promising results for a potential training aid (75). Cotton pads have also been used to absorb the odor of a fungus to prevent the transportation of fungal spores in canine testing (76). Shelf-life or longevity of these training aids have not yet been evaluated. Human scent, decomposition, and fungal odor are extremely complicated targets, making evaluation of absorption aids similarly complex. The explosive TATP, on the other hand, has a much simpler odor, and cotton absorption training aids have been evaluated for this target. TATP-cotton training aids have been successfully deployed for canine training, but have a very short lifetime. They could only be used for about 20 min before the odor was depleted (77).

Polymer-based absorption training aids provide a more even and predictable matrix than natural fibers, which can be an advantage for creating reproducible odor profiles over time. While there are currently no polymer-based absorption training aids on the market, one has been evaluated in numerous studies. Designed at the National Institute of Science and Technology (NIST), a polydimeythlsiloxane (PDMS)-based training aid has been shown through chemical evaluations to accurately simulate odor profiles for 2-ethyl-1-hexanol, cyclohexane, DNT, and TATP (78, 79). Published studies have not yet evaluated the aid in canine trials. However, the inert and absorptive capabilities of the matrix, the chemical analyses, and the steady odor release rates over time show that the training aids deserve further investigations, which are underway.

The diversity of ad/bsorption materials that exist shows how valuable they can be, as they do not require the transportation and handling of true material, and are therefore non-detonable, non-toxic, and non-infectious. They also have a larger application than dilution and encapsulation, since they can be applied to any true substance, even diseases or infectious materials. Pathogens and infectious agents cannot necessarily be diluted or encapsulated to be rendered safe, given that these methods do not negate the infectious material in any way. There may be options for processing some pathogens by autoclave or radiation for example, but these methods should be tested for each true material to ensure safety, particularly with pathogens of highest concern to public health, safety, and economic impact.

Ad/bsorption matrices, just like the matrices used for pseudos, dilutions, and encapsulations, have considerations that cannot be overlooked. Each volatile compound will interact differently with a different matrix, so one matrix cannot be assumed to function for all target substances. Even if one matrix is applicable for multiple target materials, it will provide different saturation points and diffusion rates for each material. Such transport properties will define the rates at which various volatiles are released from the matrix, and may alter the odor profile in undesired ways if left uncontrolled.

### Extraction

Liquid extraction of an odor from its source material is a process which removes chemical components of the odor from its true material using a solvent, such as the liquid pentane. Thus, the true material is removed and the remaining solvent is used for training. So far, this technique has a very limited application and has only been used to create training aids for insects and pests. This is advantageous as it omits the need to use live pests in cases where they are not native or may be undesirable. A recent publication by Moser et al. (45) demonstrated chemical similarity between the extract of one insect and the live insect. Further, they observed good canine selectivity and sensitivity (100%, *n* = 2) to the live insect following initial training on the extract. Extraction-based odors were also tested by dogs trained to detect bed bugs with a 100% response rate (80). Extraction has not been used very often in canine training, but these studies show promise in its application to create non-pseudo alternative training aids for insects.

## Considerations for Training Aid Production

There are several reasons why some pseudos and non-pseudo alternatives do not provide the desired success rate for canines. One is that the community does not yet fully understand the interaction of odor production, olfactory detection, and behavioral identification. It can therefore be difficult to determine exactly which targets to select or how a canine will perceive any given material. As Rice and Koziel (28, 58) have concluded, the abundance of a chemical in the headspace of a target is not equivalent to the odor impact. Chemical instrumentation and canine olfaction are different sensory systems with canines having a perceptual component to verification of the presence of a substance. Instruments and canines each have separate functional biases that do not attribute equivalent value to various volatile chemicals. While this challenge has been overcome for certain active odorants, it is difficult and full investigations are not always undertaken.

Another underlying cause for the lower than expected performance of alternative training aids is the tendency in olfactory work and research to underestimate matrix effects in order to focus on the odor/scent produced. While it is true that the odor is the target being identified or tracked, the matrix surrounding the material will influence that odor and determine how accurately an odor profile mimics that of the true material. Further, matrix effects can occur for each type of training aid: true material, pseudos, and non-pseudo alternatives. Whether the target material is merely wrapped for containment, or intentionally diluted, encapsulated, or ad/bsorbed, the matrix is important to consider for several reasons. First, many matrices, such as clear plastic bags, wood blocks, or nylon stockings, produce large volumes of odor which may compete with the target. Second, the target will interact with the matrix due to chemical properties of the materials. Vapor pressure, rates of evaporation and sublimation, polarity, diffusion rates, solubility, and molecular weight will each influence the transport properties of how a target moves through and is released from its matrix. There is no one matrix that is appropriate for all targets.

Finally, such information needs transparency. It has been previously noted [by Bradshaw (55), Simon and DeGreeff (69), among others] that the lack of third-party evaluations leads to confusion for practitioners. Training aids should be validated by chemical, biological, or physical analyses and behavioral evaluation in tandem in order to verify that what manufacturers see chemically is confirmed by the canine operationally. Third-party canine evaluations of training aids are essential to support chemical validations, and should clearly define the methods used. Assessments should be objective through such means as double-blind evaluations and distractor odors, plus should accurately measure and report sensitivity and selectivity [see for example (81, 82)]. Thorough reporting of methods for all types of analysis is significant for understanding the efficiency of training aids. Such due diligence is very important when considering operational teams that can encounter setbacks in training regimens without optimal equipment.

## Conclusion

The goal of this article was to provide language and discussion about canine training aids based on the available knowledge. Canine training aids can be categorized based on the level of contribution of the true material in their manufacture. True material is simply the actual target substance, whether solid, liquid, or gas. Pseudo-odors are manufactured without direct influence of the true material. Non-pseudo alternatives utilize the true material in their manufacture and render safe the true material in some manner. Within those categories, their chemical nature and function should be used for labeling. Specifically, dilution and encapsulation methods contain trace amounts of the true material that may be safely handled. Ad/bsorption and extraction methods, on the other hand, contain only the odor of the true material, rather than the material itself.

Further, the article discussed the available literature surrounding efficacy of these various forms of training aids. While true material is considered best practice for most situations, there may be challenges in the handling, transportation, storage, or the variety of available targets and odor profiles that can complicate the selection of true material. Because true material is not always available for logistical, safety, or regulatory reasons, alternative training aids can be of great value. However, these alternatives should be approached with care, as much of the discussed literature has cautioned.

## Author Contributions

All authors listed have made a substantial, direct and intellectual contribution to the work, and approved it for publication.

## Conflict of Interest

AS was employed by the company AGS Forensics, LLC. KG was employed by the company Giles Consulting, LLC. The remaining authors declare that the research was conducted in the absence of any commercial or financial relationships that could be construed as a potential conflict of interest. The handling editor declared a past co-authorship with one of the authors PW.
